# Exploring Aesthetic Outcomes and Complications in Auricular Reconstruction Utilising Autologous Cartilage: A Systematic Review and Narrative Synthesis

**DOI:** 10.7759/cureus.56345

**Published:** 2024-03-17

**Authors:** Munir Abukhder, Sam Tarassoli, Ridwanul Hassan, Elizabeth Onions, Salmane Nasri Elmi, Rhys Whelan

**Affiliations:** 1 Oral and Maxillofacial Surgery, Northwick Park Hospital, London, GBR; 2 Plastic Surgery, Morriston Hospital, Swansea, GBR; 3 Burns and Plastic Surgery, Queen Elizabeth Hospital Birmingham (QEHB), Birmingham, GBR; 4 Medicine, University of Manchester, Manchester, GBR; 5 Dentistry, King's College London, London, GBR; 6 Library, Morriston Hospital, Swansea, GBR

**Keywords:** complications’, ear aesthetics, otoplasty, ear reconstruction, plastic surgergy, auricular reconstruction

## Abstract

Auricular reconstruction remains a challenging procedure, requiring a high degree of manual dexterity and attention to detail in order to reconstruct the complex three-dimensional geometry of the ear successfully. Most techniques will rely on autologous cartilage for auricular framework fabrication, carrying a risk of donor and recipient site morbidity. The aim of this report is to investigate the complications and aesthetic outcomes associated with autologous cartilage harvest in auricular reconstruction. A systematic review protocol was registered with the International Prospective Register of Systematic Reviews (PROSPERO) and reported in accordance with the Preferred Reporting for Items for Systematic Reviews and Meta-Analyses. Comprehensive electronic search strategies for four databases were developed. Studies were screened according to the inclusion and exclusion criteria by two independent reviewers. The literature search identified 7171 articles. Filtering for relevance and duplication reduced the number of articles to 52. A total of 12,215 patients underwent auricular reconstruction utilising autologous cartilage. Indications included 11,696 patients due to microtia, 334 patients due to burns or trauma, 70 patients due to constricted ears, and 115 patients due to prominent ears. The most commonly reported donor site complications included chest wall deformities (n = 159). The most commonly reported recipient site complications included hypertrophic or keloid scars (n = 279), haematoma (n = 155), tissue expander exposure (n = 111), cartilage or framework exposure (n = 122), and cartilage framework deformation or resorption (n = 50). Although a challenging procedure, auricular reconstruction utilising autologous cartilage is possible. Exceptional aesthetic results can be achieved when performed by a skilled surgeon on appropriately selected individuals. However, the potential risks and complications associated with the procedure should be discussed with the patient and family beforehand.

## Introduction and background

While the first descriptions of auricular reconstruction were documented in the Susrata Samhita in 600 B.C., the surgical techniques in contemporary autologous auricular reconstruction have undergone significant evolution in the last century [1]. Pioneers in the field, such as Brent, Nagata, Tanzer, and Firmin, form the basis of the majority of techniques currently utilised by surgeons performing autologous ear reconstruction throughout the globe [2-5]. These techniques may vary in their number of stages, and they may also vary in their utilisation of skin expanders [6-8]. Regardless of the reconstructive technique utilised, however, the primary aim is to reproduce the natural appearance of the ear through successful ear framework fabrication and framework coverage. Nevertheless, auricular reconstruction remains a challenging procedure. The operating surgeon will need to have a high degree of manual dexterity and attention to detail in order to successfully reconstruct the complex three-dimensional geometry of the ear [9,10]. In addition to achieving a satisfactory aesthetic outcome, a successful reconstruction may also result in improvements in the patient’s psychological state, health, and quality of life, as craniofacial disfigurement, including microtia, has been reported within the literature to have a negative impact on a patient’s self-confidence and social functioning [11-14].

Regardless of the strategy or approach taken in autologous auricular reconstruction, most techniques will rely on autologous cartilage for auricular framework fabrication [3,4,15-17]. Costal cartilage remains a primary source for the fabrication of the auricular framework; however, it is not without its problems. In the paediatric patient cohort, for example, its growth will determine the feasibility and outcomes of this procedure. Additionally, autologous cartilage harvest, in general, will always carry a risk of donor site morbidity, including complications such as pain, scarring, wound dehiscence, and injury to local structures. Furthermore, the risk of graft distortion and resorption leading to poor long-term outcomes also remains an issue with autografts [18]. Although synthetic options have been described, the use of autologous tissue remains the current gold standard, despite the aforementioned complications and risks. This is primarily due to the higher biocompatibility and immunocompatibility of autologous tissue.

In this systematic review, we aim to summarise the most commonly reported donor and recipient site complications in ear reconstruction with autologous cartilage and the reported aesthetic outcomes.

## Review

Methods

Literature Search

A systematic review protocol was registered with the International Prospective Register of Systematic Reviews (PROSPERO; CRD42022300402). This review is reported in accordance with the Preferred Reporting for Items for Systematic Reviews and Meta-Analyses (PRISMA) [19]. Comprehensive electronic search strategies were developed for each database using a combination of relevant keywords and index headings. A total of four bibliographic databases were searched (Embase, MEDLINE, CINAHL Plus, and the Cochrane Central Register of Controlled Trials). The search strategy was modified so that the index headings relevant to each specific database were selected. The search strategy was peer-reviewed by an information specialist. Forward and backward citation searches were conducted on articles identified as eligible for full-text review. Full search strategies and results are contained in Supplement 1.

Duplicate papers were identified and removed in Endnote 20 before being uploaded to Rayyan systematic review software for screening. Two independent reviewers screened titles and abstracts according to the inclusion and exclusion criteria (Table 1). The remaining articles were downloaded in full-text format and re-screened. Discussion with a senior author to achieve consensus resolved any conflicts between the two reviewers.

**Table 1:** Inclusion and exclusion criteria

Inclusion criteria	Exclusion criteria
Primary research paper investigating auricular reconstruction with autologous cartilage graft	Systematic/narrative reviews, case reports, book chapters, abstracts, comments or notes
English language studies only	Primary research papers with less than 30 cases
	Non-English language papers
	Animal or cadaveric studies

Data Extraction

The following information was extracted from full-text articles onto a customised Microsoft Excel spreadsheet (Microsoft® Corp., Redmond, WA): (i) study characteristics, including author, year of publication, and sample size; (ii) patient demographics; (iii) procedure performed; (iv) cartilage graft location; and (iv) outcome data, including the complication types and rates, and satisfaction rates.

Synthesis

A meta-analysis was not considered for this review due to the heterogeneous nature of the results. However, a narrative synthesis was performed to synthesise the findings of the different studies. The results of the studies were discussed and structured into themes, depending on the aetiology and the site of cartilage harvest. This formed the framework for our narrative synthesis. All articles in this review were fully published, peer-reviewed publications. The quality and risk of bias of the studies eligible for inclusion were evaluated using the Newcastle-Ottawa Scale (NOS) [20]. Studies with NOS scores of 0-3, 4-6, and 7-8 were considered low, moderate, and high quality, respectively.

The methodological quality of this systematic review was evaluated by our team by utilising A Measurement Tool to Assess Systematic Reviews 2 (AMSTAR-2) [21]. This tool is comprised of 16 items, with seven critical items and nine non-critical items. For non-critical items, we assigned 1 point for ‘Yes’, 0.5 for ‘Partial Yes’, and 0 for ‘No’. For critical items, the score was double. The total AMSTAR-2 score was 23 points [22,23].

Results

**Figure 1:**
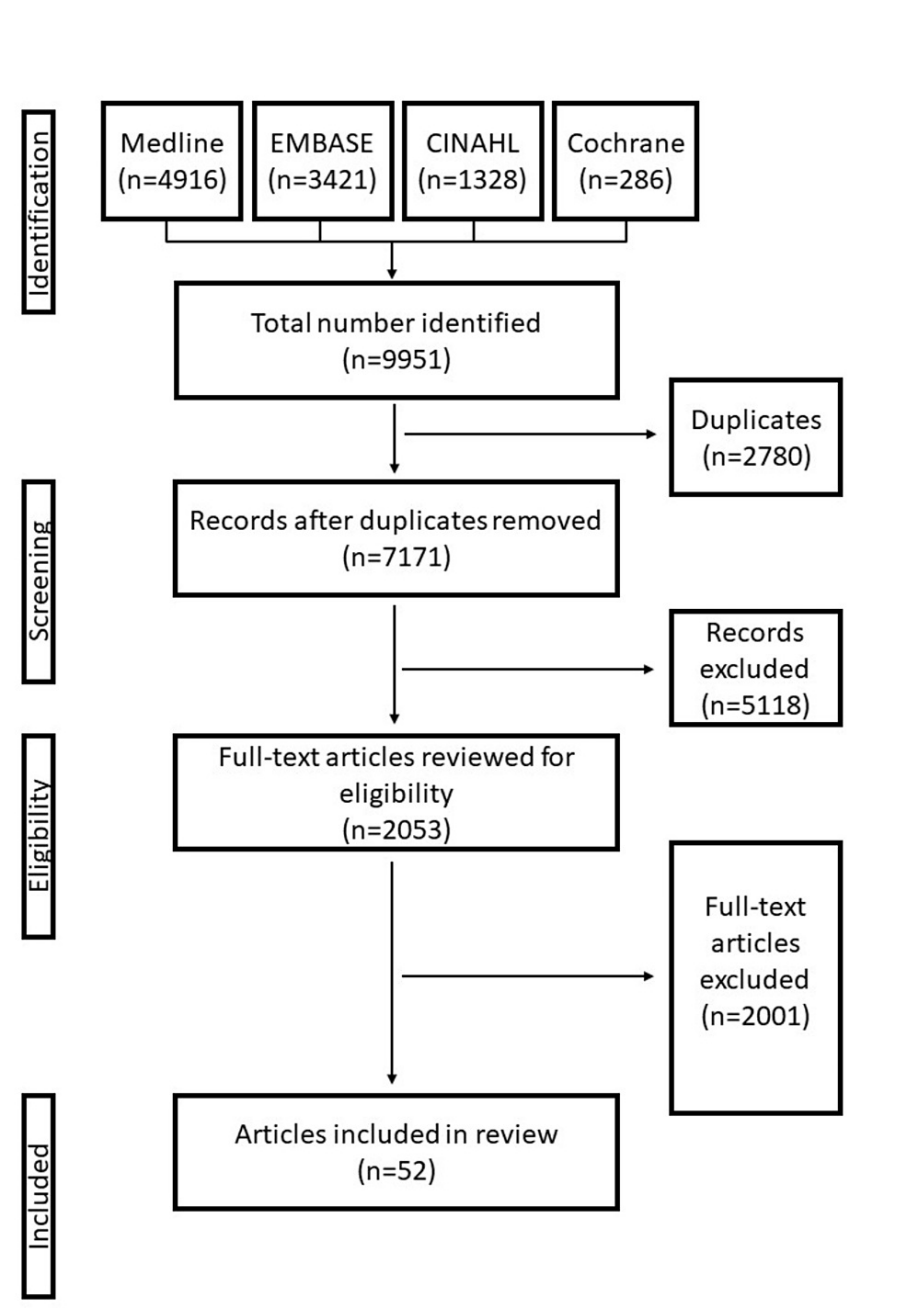
Prisma flow diagram

Following a comprehensive literature search, we identified 7171 articles. These articles were filtered for relevance and duplication, resulting in 2053 articles. A full-text assessment was then performed, which reduced the number of articles to 52. With regard to the quality of the evidence, all of the studies eligible for inclusion were considered to be of fair quality. The AMSTAR-2 score for this systematic review was 13. The PRISMA flow diagram is summarised in Figure 1.

Congenital Ear Deformities

Forty-three articles investigated the use of autogenous cartilage in auricular reconstruction secondary to congenital deformities in 11,696 patients [6,7,14,24-63]. Regarding the location of the autologous cartilage graft, costal cartilage was the most utilised donor site. Other sites included septal cartilage and conchal cartilage. Table 2 summarises the studies included that investigated the use of autologous cartilage in patients with congenital ear deformities, the NOS scores of the relevant studies, their patient demographic details, aetiology, source of cartilage, and aesthetic outcome.

**Table 2:** Congenital ear deformity studies *All ages are in years; NOS: Newcastle-Ottawa Scale score, NR: not recorded ^a^Three-point satisfaction scale (great satisfaction, satisfaction, and unsatisfaction) ^b^Four-point scale in which each score was assigned to the following: (1) poor, (2) fair, (3) good, and (4) excellent ^c^Satisfaction levels were defined as ‘‘very satisfactory,’’ ‘‘satisfactory,’’ ‘‘fair,’’ and ‘‘not satisfactory.’’ ^d^5-point ordinal scale from 1 (very poor) to 5 (very good) ^e^Satisfaction levels were defined as “satisfied,” “relatively satisfied,” and “dissatisfied” ^f^Evaluated according to 4 grades: very good, good, fair, and poor ^g^Satisfaction levels were defined as “excellent,” “good,” “fair,” and “poor” ^h^Satisfaction levels were defined as “satisfactory,” “partially satisfactory,” or “unsatisfactory” ^i^Satisfaction levels were defined as “very satisfactory,” “satisfactory,” “poor satisfactory,” or “not at all” ^j^Divided into three categories: satisfied, acceptable or unacceptable ^k^A score of 0 signifies no change in health-related quality-of-life, +100 indicates maximal improvement, and –100 indicates maximal negative impact ^l^Recorded on a five-point ordinal scale

Author (year)	NOS score	Total number of patients	Male	Female	Age *	Aetiology description	Cartilage harvest site	Aesthetic outcomes
Sun et al. [48]	7	50	31	19	Mean: 5.74 Range: 2-9	Microtia (type 1: 8, type 2: 17, type 3: 25)	Concha	Great satisfaction: 45 patients; satisfaction: 5 patients^a^
Wang et al. [61]	7	163	107	56	Mean: 8.4 Range: 6-17	Microtia (lobule-type: 124, concha-type: 39)	Costal	Modification group mean aesthetic score 3.13^b^; traditional group mean aesthetic score 3.06^b^
Dong et al. [47]	5	107	75	32	Mean: 14.4 Range: 7-27	Microtia (subtype not stated)	Costal	Very satisfactory: 80; Satisfactory: 18; Fair: 5; Not satisfactory: 4
Mazeed and Bulstrode [50]	5	363	212	151	Mean: 10.9 Range: 9-19	Microtia (subtype not stated)	Costal	The overall aesthetic score was 3.2 ± 0.62^d^
Yan et al. [39]	6	68	44	24	Range: 5-10	Microtia (type I=12, type II=37, type III=19)	Costal	Satisfied: 66 patients; Relatively satisfied: 2 patients^e^
Fu et al. [52]	5	429	N/A	N/A	Mean: 12.27 Range: 6-32	Microtia (subtype not stated)	Costal	NR
Cugno and Bulstrode [25]	5	206	N/A	NR	Median: 11.4 Range: 10-18	Microtia (concha-type: 1, lobule-type: 205)	Costal	NR
Xu et al. [26]	3	562	397	165	Range: 6-42	Microtia (all lobule-type)	Costal	NR
Tripathee et al. [49]	7	180	127	53	NR	Microtia (subtype not stated)	Costal	NR
Lee and Oh [58]	8	309	219	90	Mean: 15.1 Range: 9-56	Microtia (lobule-type: 235, concha-type: 88)	Costal	NR
Li et al. [31]	5	121	76	45	Range: 6-32	Microtia (subtype not stated)	Costal	NR
Ma et al. [57]	4	243	151	92	Mean: 18.9 Range: 6-62	Microtia (lobule-type: 174, concha-type: 65, anotia: 4)	Costal	Poor: 23; Fair: 25; Good: 163; Very good: 32^f^
Kim et al. [59]	5	50	36	14	Mean: 16.3 Range: 10-49	Microtia (concha-type: 18, lobule-type: 32)	Costal	Auricular symmetry: 47 patients achieved “excellent” and “good” outcomes; Projection: 43 patients achieved “excellent” and “good” outcomes ^g^
Xing et al. [54]	4	89	61	28	Mean: 31 Range: 24-50	Microtia (subtype not stated)	Costal	80 satisfied ^h^
Xing et al. [55]	7	54	43	11	Mean: 11.12 Range: 6-35	Microtia (lobule-type: 38, concha-type: 12, mix: 4)	Costal	50 patients and their families reported “very satisfactory” or “satisfactory” outcomes^i^
Yang et al. [32]	6	56	42	14	Range: 6-25	Microtia (lobule-type: 40, concha-type 15, anotia 1)	Costal	54 patients were satisfied with the reconstructed auricle and helix, antihelix, superior crus, and inferior crus.
Xu et al. [53]	1	390	298	92	Range: 6-40	Microtia (all lobule-type)	Costal	381 patients were satisfied with the lobule, helix, anti-helix, concha, and adjacent structures
Xu et al. [56]	4	415	324	91	Range: 6-40	Microtia (sausage-type: 185, lobule-type: 78, concha-type: 152)	Costal	407 patients were satisfied with the helix, crus helicis, and adjacent structures
Li et al. [29]	3	91	68	23	Mean: 14 Range: 6-31	Microtia (subtype not stated)	Costal	Authors report patients and their relatives were satisfied with the results. No reporting measure utilised
Zhang et al. [30]	4	58	42	16	Mean: 13 Range: 6-42	Microtia (all hunter type 3)	Costal	Satisfactory: 54 ears; Acceptable: 5 ears
Cui et al. [28]	7	72	57	15	N/A	Microtia (all lobule-type)	Costal	Average satisfaction rate for auricle substructures was 74%
Kim et al. [33]	5	48	NR	NR	NR	Microtia (subtype not stated)	Costal	NR
Sakamoto et al. [38]	6	122	88	34	Mean: 9.8 Range: 7-18	Microtia (subtype not stated)	Costal	NR
Zhou et al. [51]	5	103	59	44	Mean: 21.5 Range: 16-43	Microtia (lobular-type: 54, concha-type: 49)	Costal	Satisfactory: 84 patients; acceptable: 14 patients; unacceptable: 5 patients ^j^
Soukup et al. [14]	7	55	27	28	Mean: 12 Range: 9-17	Isolated microtia/anotia: 40 syndromic microtia: 15	Costal	Mean total Glasgow Benefit Inventory score was 48.1, with significant improvements seen in all three subscales^k^. Mean integration score of 3.8^l^. Mean aesthetic auricular unit reconstruction score of 3.4^l^.
Zhao et al. [27]	5	1300	860	440	Mean: 13.5 Range: 6-25	Microtia (subtype not stated)	Costal	Total satisfaction rate was 85.5%
Chin et al. [35]	5	166	N/A	N/A	Mean: 16 Range: 6-42	Microtia (all lobule-type)	Costal	162 patients were satisfied with the reconstructed auricle
Chin et al. [46]	3	125	86	39	Range: 6-50	Microtia (sausage-type: 69, lobule-type: 17, concha-type: 40)	Costal	The “great majority” of patients were satisfied with the three-dimensional configurations and shape of the auricle.
Zhang et al. [60]	4	350	254	96	Range: 5.5-50	Microtia (lobule-type: 278, concha-type:72)	Costal	288 patients were satisfied with the outcomes
Steffen et al. [63]	5	60	NR	NR	Median: 20 Range: 12-58	Microtia (subtype not stated)	Costal	52 patients reported they integrated the reconstructed auricle into their body concept
Jiang et al. [7]	5	3332	1741	1591	Range: 5-11	Microtia (subtype not stated)	Costal	NR
Dashan et al. [45]	6	342	NR	NR	NR	Microtia (lobule-type: 329, concha-type: 22, small concha-type: 13, anotia: 2)	Costal	Excellent: 21; Good 75; Fair: 16; Poor: 6^g^
Jiang et al. [36]	2	332	241	91	Range: 5-33	Microtia (subtype not stated)	Costal	All the reconstructed ears had a satisfactory three-dimensional configuration
Pan et al. [44]	4	368	254	114	NR	Microtia (subtype not stated)	Costal	“Most” patients were satisfied
Cho et al. [43]	3	125	69	56	Range: 7-53	Microtia (lobule-type: 94, concha-type: 42)	Costal	Acceptable: 118; blunted convolution: 6; deformation: 4
Osorno [62]	5	276	NR	NR	NR	Microtia	Costal	NR
Cho et al. [42]	5	36	23	13	Range: 7-51	Microtia (lobule-type: 17, concha-type: 20)	Costal	Acceptable: 33; Lack of detail: 3
Madura et al. [40]	1	45	25	20	Mean: 9.2 Range: 4-16	Microtia (subtype not stated)	Costal	NR
Di Mascio et al. [24]	3	35	22	13	Range: 8-40	Microtia (subtype not stated)	Costal	NR
Park [6]	3	145	112	33	Range: 10-48	Microtia (lobule-type: 137, concha-type: 6, scapha-type: 1)	Costal	NR
Hata et al. [41]	5	38	25	13	N/A	Microtia (complete hypoplasia 14, conchal remnant 13, constricted 11)	Costal	NR
Park [37]	4	51	33	18	NR	Microtia (conchal remnants: 35, scapha remnants: 7, lop-ear deformities: 7, absence of more than the total earlobe: 3)	Costal: 31 Conchal: 3 Septal 1 conchal and costal: 11 conchal and septal: 6	NR
Yanai et al. [34]	3	166	NR	NR	NR	Microtia (subtype not stated)	Costal	NR

Various approaches were taken to evaluate the satisfaction and aesthetic outcome of auricular reconstruction. A total of 1664 patients were satisfied with their reconstructed ears. The study by Sun et al. utilised a three-point satisfaction scale (great satisfaction, satisfaction, and unsatisfaction). In their study, 45 patients were very satisfied, and 5 patients were satisfied [48]. The study by Cui et al. utilised a five-point Likert-type scale ranging from 1 (very unsatisfied) to 5 (very satisfied) to assess satisfaction [28]. The average satisfaction rate for auricle substructures was 74%, with their patient cohort reporting the highest level of satisfaction with the helix and the lowest level of satisfaction with the tragus. The study by Xing et al. interviewed subjects to assess their satisfaction as “very satisfactory," "satisfactory," “poor satisfactory," or “not at all.” In their study, 50 patients and their families reported that the outcomes were either satisfactory or very satisfactory [55]. In the study by Zhou et al., 84 patients considered the outcome satisfactory, 14 patients stated it was acceptable, and 5 patients stated it was unacceptable [51]. In the study by Wang et al., the team modified the conventional crescent cartilage block by sculpting a concavity at its posterior surface, aiming to improve the retro-auricular contour [61]. They utilised a four-point scale in which each score was assigned to the following: (1) poor, (2) fair, (3) good, and (4) excellent. The mean aesthetic scores for ear projection in the modification and traditional groups were 3.13 and 3.06, respectively. The mean aesthetic scores for cranioauricular sulcus in the modification and traditional groups were 2.51 and 2.9, respectively.

Donor site complications that arose included chest wall deformities in 159 cases, pneumothorax in two cases, infection in 19 cases, and hypertrophic scarring in 10 cases. Various complications arose within the recipient site. Haematoma developed in 155 cases, and seroma developed in 8 cases. Exposure to framework/cartilage occurred in 102 cases, of which 14 were due to localised avascular necrosis and 17 were due to infection. Cartilage resorption/distortion occurred in 63 cases. Flap/graft/skin necrosis occurred in 128 cases, and operative site infection occurred in 56 cases. Scar-related complications, including hypertrophic or keloid scars, arose in a total of 279 cases. With regards to tissue expander complications, exposure to expander occurred in 111 cases, and rupture occurred in one case. Graft failure was only reported in two cases.

Trauma and Burns Cohort

Eight articles investigated the use of autologous cartilage in ear reconstruction following trauma and burn injuries [62-69]. A total of 240 patients and 94 patients underwent auricular reconstruction following trauma and burns, respectively. Of these patients, autologous costal cartilage was the most commonly harvested site for reconstruction, followed by autologous conchal cartilage. Table 3 summarises the studies included that investigated the use of autologous cartilage in patients with ear deformities secondary to trauma or burn, their NOS scores, their patient cohort demographic details, aetiology, source of cartilage, and aesthetic outcome.

**Table 3:** Trauma and burns studies *All ages are in years; NOS: Newcastle-Ottawa Scale score; NR: not recorded

Author	NOS score	Total number of patients	Male	Female	Age*	Aetiology (number of patients)	Cartilage harvest site	Aesthetic outcomes
Li et al. [65]	4	60	37	23	Mean: 31; range: 20-53	Trauma = 60	Conchal: 5; Costal: 55	Satisfied: 55 patients
Pearl et al. [69]	2	50	41	9	Mean: 26; range: 19-46	Trauma = 50	Costal	NR
Xiaobo et al. [68]	6	91	7	14	Mean: 21.3; range: 9-45	Trauma =91	Costal	Doctor assessment: Excellent: 25; Good: 48
								Patient/parent assessment: Excellent: 28; Good 46
Steffen et al. [63]	5	8	7	1	Median: 31.5; range: 16-52	Trauma = 8	Costal	All 8 patients integrated the reconstructed auricle into their body concept
Osorno [62]	5	15	NR	NR	NR	Acquired defects = 15	Costal	NR
Kobus et al. [67]	5	12	9	3	Mean for burns patients: 32	Burn = 4	Costal	NR
					Mean for trauma patients: 24	Trauma = 8		
Park et al. [66]	4	28	NR	NR	NR	Trauma = 20	Costal	NR
						Burn = 8		
Pensler et al. [64]	4	70	NR	NR	NR	Burn = 70	Concha	Good-to-excellent results (3.5±1.0 per ear)
							Costal	Fair results (2.1±0.8 per ear)

Only four articles commented on the aesthetic outcome of their patient cohort. Pensler et al. gave a numerical score between 1 and 4, depending on the degree of improvement, appearance, and projection of the helix post-operatively (1 = poor, 2 = fair, 3 = good, and 4 = excellent). Click or tap here to enter text. The conchal cartilage graft yielded good-to-excellent results (3.5 ± 1.0 per ear), and the rib cartilage graft yielded fair results (2.1 ± 0.8 per ear) [64]. In the study by Xiaobo et al., aesthetic assessment was conducted by both the doctor and the patient/parent. Twenty-five ears showed excellent results, and 48 ears showed good results on the doctor's assessment. Twenty-eight ears showed excellent results, and 46 ears showed good results on the patient/parent assessment [68]. In the study by Steffen et al., all eight trauma patients reported to have integrated the reconstructed auricle into their body concept, either well or very well [63]. Finally, in the study performed by Li et al., 55 patients were satisfied with the aesthetic outcome. The dissatisfaction of the remaining patients was due to the following: unnatural junction between graft framework and residual ear stump (n = 2), width (n = 1), and minor framework exposure secondary to skin necrosis (n = 1) [65].

The following complications were reported in our analysis: infection (n = 3), exposed framework (n = 3), haematoma (n = 1), post-operative oedema (n = 8), hypertrophic scarring (n = 2), facial nerve injury (n = 1), and flap necrosis (n = 7). Three articles included non-trauma patients in their analysis of post-operative complications. Therefore, it was not possible to distinguish the aetiology of the injury in the patients that had developed the complication [62,66,67].

Constricted Ear Cohort

One article investigated the use of conchal cartilage in patients with constricted ears [70]. A total of 139 patients (63 males and 76 females) underwent ear reconstruction. The following operative techniques were performed: antihelical tubing, concha cartilage grafting, tumbling concha cartilage flap, antihelical wrapping, or helical expansion. The authors also report using the mastoid hitch procedure as an adjunctive technique in all types of constricted ears when prominent ear deformities are combined. The authors graded their patient cohort into four classifications using the antihelical tubing test and the scapha-helix push test. This classification system helped guide the surgical management plan for each patient. The aesthetic outcome was assessed using a four-point Likert scale (1 = poor, 2 = fair, 3 = good, and 4 = excellent). The following aspects were considered in the assessment: symmetry, size, shape, and setback of the scapha-helix portion. A total of 144 ear cases were followed for 1 month to 14 years (average = 13.2 months). The average aesthetic outcome score was 3.3 overall (type I = 3.8, type II = 3.4, type III = 2.5, and type IV = 3.5). The following complications were reported: infection = 1, venous congestion = 4, scar contracture at the recipient site = 5, and irregular postauricular surface = 2.

Prominent Ear Cohort

Park et al. investigated the use of conchal cartilage in patients with prominent ears [71]. A total of 66 patients (27 males and 39 females) underwent otoplasty. The following complications were reported at the recipient site: asymmetry = 2, relapse = 1, mild irregular or prominent post-auricular surface = 3, stiff ear = 2, and tingling = 1. A hypertrophic scar in one patient was the only reported complication at the recipient site.

The study by Firmin et al. was a retrospective review of 49 patients who underwent ear reconstruction following complications of otoplasty for prominent ears [72]. Conchal cartilage was harvested in 8 patients, and costal cartilage was harvested in 41 patients. Three patients required a temporal fascia flap in order to cover the cartilaginous framework, as the local skin was insufficient. This study did not report complications following ear reconstruction.

Discussion

Although autologous costal cartilage harvesting has proven to be a reliable technique for auricular reconstruction, the procedure is not without its difficulties. The process of harvesting and fabricating an appropriate framework is a meticulous procedure, requiring careful attention to detail and surgical experience within the field. Various fabrication techniques have been developed in order to achieve a satisfactory appearance for the reconstructed auricle. However, regardless of the technique utilised, there are important characteristics that will need to be reconstructed to obtain a natural appearance, such as a prominent helix, a deep conchal cavity, an external meatus covered by a tragus, and an antihelix. Many factors will influence the chosen surgical management plan for the patient. For example, the age at which to undertake the procedure remains relatively controversial, as the costal cartilage must be suitable for harvest. Six years is believed to be the youngest age at which a patient would be deemed ready to undertake the surgery due to the limited bulk of cartilage prior to this age [73]. Additionally, the state of the patient’s rib should be taken into consideration, for example, the size, length, and shape of the costal cartilage. Furthermore, the psychological well-being of the patient can be negatively impacted if the surgery is delayed [74,75].

Costal cartilage is usually harvested from the sixth, eighth, or ninth ribs to carve the framework [39]. The degree of donor site morbidity will depend on the amount of cartilage harvested. For example, harvesting a single costal cartilage will unlikely cause significant thoracic wall deformity; however, harvesting two or more costal cartilages that bridge the gap between the ribs and sternum may cause a visible thoracic wall deformity. In the studies performed by Thomson et al. and Ohara et al., the incidence of chest wall deformity post-cartilage harvest reported was roughly 25% and 12.5%, respectively [76,77]. In this review, Dashan et al. reported an incidence of chest wall deformity of roughly 46% [45]. They attributed this high complication rate to how thin their patient cohort was and that they operated on patients between the ages of 5 and 10 years old. Their chest wall deformity rate was similar to that of Ohara et al., and they, too, harvested costal cartilage grafts from patients younger than 10 years old. Dashan et al. believe that due to the small chest circumference of their patient cohort and their young age, chest wall deformities are more obvious. Additional complications that may arise as a result of the costal cartilage harvest include pneumothorax and atelectasis. In the study performed by Thomson et al., the incidence of pleural defects in patients post-costal cartilage harvesting was roughly 22% of cases [76]. Attempts have been made to remedy the development of thoracic wall defects arising from harvesting costal cartilage, such as returning excess costal cartilage to the chest. However, deformation of the chest wall still occurred [36]. However, Kawanabe and Nagata highlighted that the key to preventing chest wall deformities was preserving the perichondrium at the donor site. In his study of 273 autologous costal cartilage harvests, he reported no chest wall deformities by adhering to this principle [78].

Various materials have been proposed for costal cartilage fixation when performing autologous auricular reconstruction. Wires are a common material utilized when fixing the auricular frame. However, the surgeon must ensure the wires are buried well within the cartilage when choosing this fixation material, thus reducing the risk of exposure [38]. Despite this, wire exposure remains a common risk in certain scenarios, for example, thinning of the overlying skin, inflammation of the recipient site, or cartilage resorption. An alternative to wires includes the use of non-absorbable monofilament sutures. However, this material tends to become loose at the ligature, thus posing a similar risk of exposure [79]. Absorbable sutures are another alternative; however, cartilage regression may occur when the tensile strength begins to diminish [38].

Various alternatives to autologous costal cartilage have been suggested in the literature. Auricular reconstruction, combined with alloplastic reconstruction, has gained popularity in recent years. It not only avoids donor site morbidity from cartilage harvest, but it also allows reconstruction to occur at an earlier age as adequate growth of rib cartilage is no longer a concern [80]. An example of an alloplastic material is Medpor (Stryker, USA), which is a synthetic, biocompatible, porous polyethylene implant [81]. Looking to the future, the 3D bioprinting field is an ever-expanding area of research. Although still in its infancy, 3D bioprinting may offer an alternative autologous tissue harvest by producing complex native-like tissue constructs and by controlling the assembly of the construct at the nano-, micro-, and macroscopic levels [82]. This would remove the need for autologous tissue harvest and transform reconstructive surgery [83,84]. However, concerns exist regarding the adoption of regenerative medical interventions into clinical practice, primarily with regard to the safety of the cells for therapeutic use and also the cost and clinical effectiveness of this treatment modality [85,86].

Limitations

We acknowledge the limitations of this review. The heterogeneous nature of the studies reduced the generalisability of our results. Variables such as the experience of the surgeon, the surgical approach and technique, aetiology, patient age, and the type and quantity of cartilage harvested did not allow for an accurate comparison to be performed between the studies. Additionally, not all studies reported post-operative complications in both donor and recipient sites; therefore, the incidence of complications is likely to be higher. Furthermore, the method by which aesthetic outcomes were reported varied across the studies included in this review. This lack of standardisation further increased the degree of heterogeneity. Finally, the majority of the studies within our review did not include a comparator group. This would likely reduce their degree of internal validity and, therefore, reduce the generalisability of our results.

## Conclusions

Auricular reconstruction utilising autologous cartilage remains a viable procedure. The attainment of outstanding aesthetic results hinges on appropriate training, case availability, and meticulous patient selection. Despite its challenges, the procedure unveils its potential for transformative outcomes. It is imperative, however, that the surgeon engage in proactive discussions with both the patient and their family, transparently outlining the potential risks and complications associated with the process. By fostering an informed decision-making environment, these preoperative conversations empower individuals to navigate the complexities of auricular reconstruction, ensuring a collaborative and patient-centred approach to the challenges and rewards this intricate procedure entails.
